# Three-Year Improvements in Weight Status and Weight-Related Behaviors in Middle School Students: The Healthy Choices Study

**DOI:** 10.1371/journal.pone.0134470

**Published:** 2015-08-21

**Authors:** Karen E. Peterson, Jennifer L. Spadano-Gasbarro, Mary L. Greaney, S. Bryn Austin, Solomon Mezgebu, Anne T. Hunt, Emily A. Blood, Chrissy Horan, Henry A. Feldman, Stavroula K. Osganian, Maria F. Bettencourt, Tracy K. Richmond

**Affiliations:** 1 Department of Nutritional Sciences, School of Public Health and Center for Human Growth and Development, University of Michigan, Ann Arbor, Michigan, United States of America; 2 Department of Nutrition, Harvard T.H. Chan School of Public Health, Boston, Massachusetts, United States of America; 3 Department of Social and Behavioral Sciences, Harvard T. H. Chan School of Public Health, Boston, Massachusetts, United States of America; 4 Center for Community-Based Research, Dana Farber Cancer Institute, Boston, Massachusetts, United States of America; 5 Department of Kinesiology, University of Rhode Island, Kingston, Rhode Island, United States of America; 6 Division of Adolescent and Young Adult Medicine, Department of Medicine, Boston Children’s Hospital, Boston, Massachusetts, United States of America; 7 Nutrition Program North East Region, United States Department of Agriculture (USDA), Boston, Massachusetts, United States of America; 8 Hunt Consulting Associates, Logan, Utah, United States of America; 9 Clinical Research Center, Boston Children’s Hospital and Harvard Medical School, Boston, Massachusetts, United States of America; 10 Obesity Prevention Program, Department of Population Medicine, Harvard Medical School and Harvard Pilgrim Health Care Institute, Boston, Massachusetts, United States of America; 11 Massachusetts Department of Public Health, Boston, Massachusetts, United States of America; University of Westminster, UNITED KINGDOM

## Abstract

**Introduction:**

Few dissemination evaluations exist to document the effectiveness of evidence-based childhood obesity interventions outside the research setting.

**Objective:**

Evaluate Healthy Choices (HC), a multi-component obesity prevention program, by examining school-level changes in weight-related behaviors and weight status and the association of implementation components with odds of overweight/obesity.

**Methods:**

We compared baseline and Year 3 school-level behavioral and weight status outcomes with paired t-tests adjusted for schools’ socio-demographic characteristics. We used generalized estimating equations to examine the odds of overweight/obesity associated with program components.

**Setting/Participants:**

Consecutive sample of 45 of 51 middle schools participating in the HC program with complete baseline and follow-up survey data including a subsample of 35 schools with measured anthropomentry for 5,665 7^th^ grade students.

**Intervention:**

Schools developed a multi-disciplinary team and implemented an obesity prevention curriculum, before and after school activities, environmental and policy changes and health promotions targeting a 5-2-1 theme: eat ≥ 5 servings/day of fruits and vegetables (FV), watch ≤ 2 hours of television (TV) and participate in ≥ 1 hours/day of physical activity (PA) on most days

**Main Outcome Measures:**

1) School-level percent of students achieving targeted behaviors and percent overweight/obese; and 2) individual odds of overweight/obesity.

**Results:**

The percent achieving behavioral goals over three years increased significantly for FV: 16.4 to 19.4 (p = 0.001), TV: 53.4 to 58.2 (p = 0.003) and PA: 37.1 to 39.9 (p = 0.02), adjusting for school size, baseline mean age and percent female, non-Hispanic White, and eligible for free and reduced price lunch. In 35 schools with anthropometry, the percent of overweight/obese 7^th^ grade students decreased from 42.1 to 38.4 (p = 0.016). Having a team that met the HC definition was associated with lower odds of overweight/obesity (OR = 0.83, CI: 0.71–0.98).

**Conclusions and Relevance:**

The HC multi-component intervention demonstrated three-year improvements in weight-related behaviors and weight status across diverse middle schools. Team building appears important to the program’s effectiveness.

## Introduction

Persistent trends in pediatric obesity underscore the need to identify evidence-based strategies to reach large numbers of children and adolescents.[1] Public health agencies and expert panels have advocated multi-component, school-based programs [2–4] that can address the complex influences on weight-related behaviors using a systems-level approach to primary prevention of obesity. [5–11]

Evidence for the efficacy of multi-component, school-based obesity prevention programs largely relies on findings from randomized controlled trials (RCT).[10, 12, 13] While the RCT remains the gold standard to ensure internal validity, modest sample sizes and homogenous study populations may limit generalizability of findings.[14, 15] The staffing and resources required for implementation also may be difficult to sustain after the trial period, potentially diluting the effect size. Involving school personnel in the implementation of programs may lead to improved sustainability; however, qualitative research has demonstrated the need for supports such as faciliators and defined teams for school personnel-led implementation strategies.[16–18]Public health efforts to mount child obesity prevention programs require additional, practice-based evidence to establish external validity and ensure contextual relevance once implemented in real-world settings.[19] Nevertheless, few studies have employed evaluation designs that can inform the institutionalization of health promotion interventions.[20, 21]

This study built on a prior RCT showing a reduced odds of obesity among girls in public middle schools implementing Planet Health, an interdisciplinary curriculum promoting healthy dietary and physical activity behaviors.[22] We used a time-series design to examine whether Healthy Choices (HC), a widely disseminated systems-level, obesity prevention program including Planet Health and other intervention components, improved school-level weight-related behaviors and weight status over three years. A secondary objective used a process implementation evaluation to identify HC program components (i.e., Planet Health curriculum, before and after school programs, environmental and policy assessments and changes, school-wide promotions, and the development of a team) associated with odds of overweight and obesity at follow-up.

## Methods

### Setting and Subjects

Massachusetts Healthy Choices (HC) was a school-based, multi-component intervention aimed at improving students’ dietary and physical activity behaviors and weight status, implemented in 128 middle schools from 2004–2009 by the Massachusetts Department of Public Health (MDPH) in collaboration with Blue Cross Blue Shield of Massachusetts (BCBSMA). Sixty-nine schools initiated their participation in academic year 2004–2005, 51 schools in 2005–2006 and eight in 2006–7. Program components were: 1) Planet Health interdisciplinary curriculum, [22, 23] 2) before- and after-school activities, 3) environmental and policy assessment and changes, 4) school-wide health promotion campaign, and 5) development of a multi-disciplinary implementation team, including a school HC coordinator. All intervention activities promoted a 5-2-1 theme targeting three behavioral goals:

5: Consume 5-9 servings of fruits and vegetables per day
2: Limit screen time to no more than 2 hours per day
1: Participate in at least 1 hour of physical activity on most days

Four regional coordinators at MDPH provided on-going technical assistance by telephone and in-person visits to schools’ HC coordinators and HC team members throughout the school year and at annual networking conferences for staff from all participating schools. Each school received funding for three years of implementation ($5,000, Year 1; $3,000 Year 2; $1,000 Year 3).

The selection of HC components was based on evidence from a range of evaluation designs.

Planet Health, an interdisciplinary middle school curriculum, targeted four key behavioral changes consistent with the 5-2-1 goals [22, 23]: reducing television viewing, increasing moderate to vigorous physical activity, decreasing consumption of foods high in fat and saturated fat, and increasing consumption of fruits and vegetables.[23] The interdisciplinary curriculum incorporated intervention messages into core school subjects such as language arts, math, social sciences as well as into physical education (PE) classes. In a group-randomized RCT, Planet Health, significantly reduced television viewing in girls and boys in intervention schools, compared with students in control schools.[22] Among girls but not boys, participation in Planet Health was associated with increased fruit and vegetable intake, a lower change in energy consumption and halved the odds of obesity among girls over two years.[22] Reduced television viewing time mediated the effect of Planet Health on obesity among girls. The Planet Health curriculum was adapted for Healthy Choices by lowering the requirements from four to two lessons within each of the four subject areas. This change was well accepted in a community-based participatory research study of diffusion of Planet Health in Massachusetts public schools.[24]

The before or after school program components of Healthy Choices were derived from the Healthy Choices Before and After School (HCBAS) program, a Massachusetts middle-school program designed to promote nutrition and physical activity initiatives in the before- and after-school setting. It was implemented in nine Massachusetts middle schools and was found to improve girls’ nutrition knowledge and stabilize girls’ mean BMI from baseline to follow-up compared with an increase in BMI in the comparison group.[25]

Schools’ policy and environmental assessment and changes were guided by the School Health and Safety Policy and Environments’ module of the Centers for Disease Control and Prevention (CDC) School Health Index.[26] The development of a HC team with a key leader and supporting activities were informed by a qualitative rapid assessment study we conducted in 2004–2005.[17] Schools were given flexibility to choose specific activities to to satisfy the requirements for implementing the HC intervention.[18] Specifically, each school was required to do two of four Planet Health modules per subject, choose one before or after school activity, choose one policy or environmental change, and one school-wide promotion per year. Each school was also encouraged to develop a multi-discipline implementation team.

Of the 51 schools initiating HC in 2005–6, 47 volunteered to participate in a prospective evaluation. The demographic characteristics of the participating schools were similar to those of all MA middle schools. Healthy Choices participating schools had 27.6% of students eligible for free and reduced lunch, 70.3% self-identified as non-Hispanic white v. 25.2% and 66.9% in the broader population of MA middle schools. We employed a time-series design consistent with school-based surveillance protocols widely used in the United States. A complete census of 6^th^, 7^th^, and 8^th^ grade students in participating schools completed anonymous, self-administered surveys in classrooms at baseline in Fall 2005 and at follow-up in Spring 2008. Heights and weights of students in 7^th^ grade were measured by school nurses in 35schools that volunteered to collect and report anthropometry using standard protocols.[27, 28] The demographic characteristics of the 35 schools who provided measured anthropometric data were also similar to those that did not. Schools providing measured heights and weights were 49.7% female, 69.9% white, with 28.9% eligible for free and reduced lunch and a mean age of 12.8 years; schools that did not provide anthropometry were 48.3% female, 71.6% white with 22.9% eligible for free and reduced lunch and with mean age 12.6 years.

Schools’ HC coordinators completed annual progress reports of types and intensity of activities implemented during the academic year. One school dropped out of the evaluation in Year 1 and one dropped out in Year 2. The analytic sample for three-year changes in self-reported 5-2-1 behavioral outcomes comprised data on 6^th^, 7^th^, and 8^th^ grade students, aggregated at the school level in the 45 schools. In 35 of 42 schools that provided heights and weights measured in the same season survey data were collected at baseline and follow-up, 5,665 students in 7^th^ grade were the sample for calculating three-year changes in percent overweight and obese and estimating the impact of intervention components on weight status.

#### Ethics statement

Study protocols were approved by the institutional review boards of MDPH, the Harvard T. H. Chan School of Public Health and Boston Children’s Hospital. Each of the 47 participating schools invited students to participate. Passive consent was obtained from parents and assent from the student participants. This consent procedure was approved by the institutional review boards of both Boston Children’s Hospital and the Harvard T. H. Chan School of Public Health.

### Main Outcomes

#### Behavioral outcomes

Awareness of the 5-2-1 message was queried with a single item: “Have you heard of the phrase ‘5-2-1’ which is part of the Healthy Choices program (yes/no)?” Measures of behavioral outcomes were adapted from Youth Risk Behavior Survey (YRBS) measures [29] and evaluations of Planet Health.[22] Daily fruit and vegetable (FV) intakes were estimated with three items asking times in past seven days the student drank or ate 100% fruit juices, fruit and vegetables (not counting potatoes). Daily hours of television viewing (TV) were calculated from two questions asking: “On an average school (or weekend) day, how many hours do you watch TV or videos?” Two additional items assessed hours spent playing electronic games and using the Internet for fun, on school and weekend days. Daily hours were calculated for TV viewing and total screen time. Physical activity (PA) was estimated from a single item asking the student the number of days in the past seven days s/he was “physically active for at least 60 minutes per day”. Categorical measures were created for each 5-2-1 behavioral target.

#### Weight status

Biologically implausible values for measured weights and heights were removed using CDC criteria [28]. BMI was calculated as weight (kg)/ height (m)^2^. Overweight was classified as BMI greater than the 85^th^ percentile and less than or equal to the 95^th^ percentile and obesity as >95^th^ percentile of sex- and age-specific reference growth curves.[30]

#### School socio-demographic characteristics

Students reported grade, age in months and race/ethnicity in Fall 2005. School size was reported from mid-year administrative records by principals and percent of students in schools eligible for free and reduced price school lunch in 2005–2006 was obtained from the National Center for Educational Statistics.[31]

#### HC implementation dose

We used information from the schools’ Progress Reports completed in the Spring 2007, reflecting extent of implementation through Year 2, to estimate dose of HC program components delivered prior to Year 3 follow-up. We used the counts of individual Planet Health variables (lessons taught, micro-units taught, teachers teaching Planet Health, student lesson-exposures (for example, 100 students taught two lessons = 200 student lesson-exposures), school-wide promotions, before- and after-school activities and environmental and policy changes addressing the 5-2-1 theme. We documented the number of outside partners in Year 2 and summed expenditures of HC funds provided to the schools across Years 1–3. A school was classified as meeting the definition of a team if the school’s HC coordinator responded yes to each of four questions: 1) Does your school’s HC team have a shared vision regarding your school’s HC program? 2) As a group, did your school’s HC team develop short-term goals related to your school’s HC program? 3) As a group, did your school’s HC team develop any long-term goals related to your school’s HC program? 4) Does your school’s HC team meet as a group?

### Statistical Analysis

Because the school was the unit of intervention, we estimated change in outcomes from Fall 2005 to Spring 2008 at the school level. We computed means and frequencies of behavioral outcomes within each school, then the mean, standard deviation (SD) and range of the school-level means across 45 schools. We computed percent overweight and obese based on anthropometry of 7^th^ grade students in 35 schools. The student’s paired t-test was used to test for the significance of differences from baseline to follow-up, adjusted for school size and baseline percent female, white, and eligible for free and reduced price lunch and mean age.

To examine the relationship of dose variables with overweight and obesity, we used Generalized Estimating Equations in order to maximize statistical power, using procedures described elsewhere [30].[32] We evaluated multicollinearity among HC dose variables and omitted the number of Planet Health lessons taught given its high correlation with the number of teachers teaching Planet Health lessons. We estimated the odds of overweight or obesity (BMI ≥ 85%tile) and of obesity (BMI >95%tile) associated with the Year 2 counts of each HC dose variables, adjusted for baseline weight status, school characteristics and total expenditure of HC funds. We estimated odds of overweight and obesity in multivariable models using stepwise backward elimination procedures to develop a parsimonious model, retaining HC implementation variables significant at p ≤ 0.10.

## Results

The mean age of students was 12.8 years. As shown in Table 1, the sample was diverse racially/ethnically. A mean of 27.6% of students were eligible for free and reduced-price lunch, but percentages ranged widely across schools, from 2.4 to 85.6%.

**Table 1:** Baseline Socio-demographic Characteristics of Schools Participating in Massachusetts Healthy Choices (n = 45).

	Mean % Across Schools (SD)		Range
**Sex**			
Female	50.6	(4.4)	[34.4–60.3]
Male	49.4	(4.4)	[39.7–65.6]
**Grade**			
6th	32.2	(16.0)	[0.0–100.0]
7th	34.7	(9.8)	[0.0–58.1]
8th	32.5	(12.1)	[0.0–55.1]
**Race/ethnicity**			
Non-Hispanic White	70.3	(23.1)	[4.3–95.4]
Asian	3.6	(4.6)	[0.0–20.1]
Asian and non-Hispanic White	0.7	(0.8)	[0.0–3.7]
Non-Hispanic Black	7.4	(12.7)	[0.0–66.0]
Non-Hispanic Black and non-Hispanic White	0.8	(0.8)	[0.0–2.9]
Hawaiian/ Pacific Islander	0.5	(0.6)	[0.0–3.0]
American Indian/ Alaska Native	1.3	(1.1)	[0.0–6.3]
Latino	8.5	(11.7)	[0.0–67.0]
Missing Ethnicity	7.1	(5.3)	[1.5–30.1]
**Students eligible for free and reduced-price school lunch**	27.6	(20.0)	[2.4–85.6]

**Fig 1:**
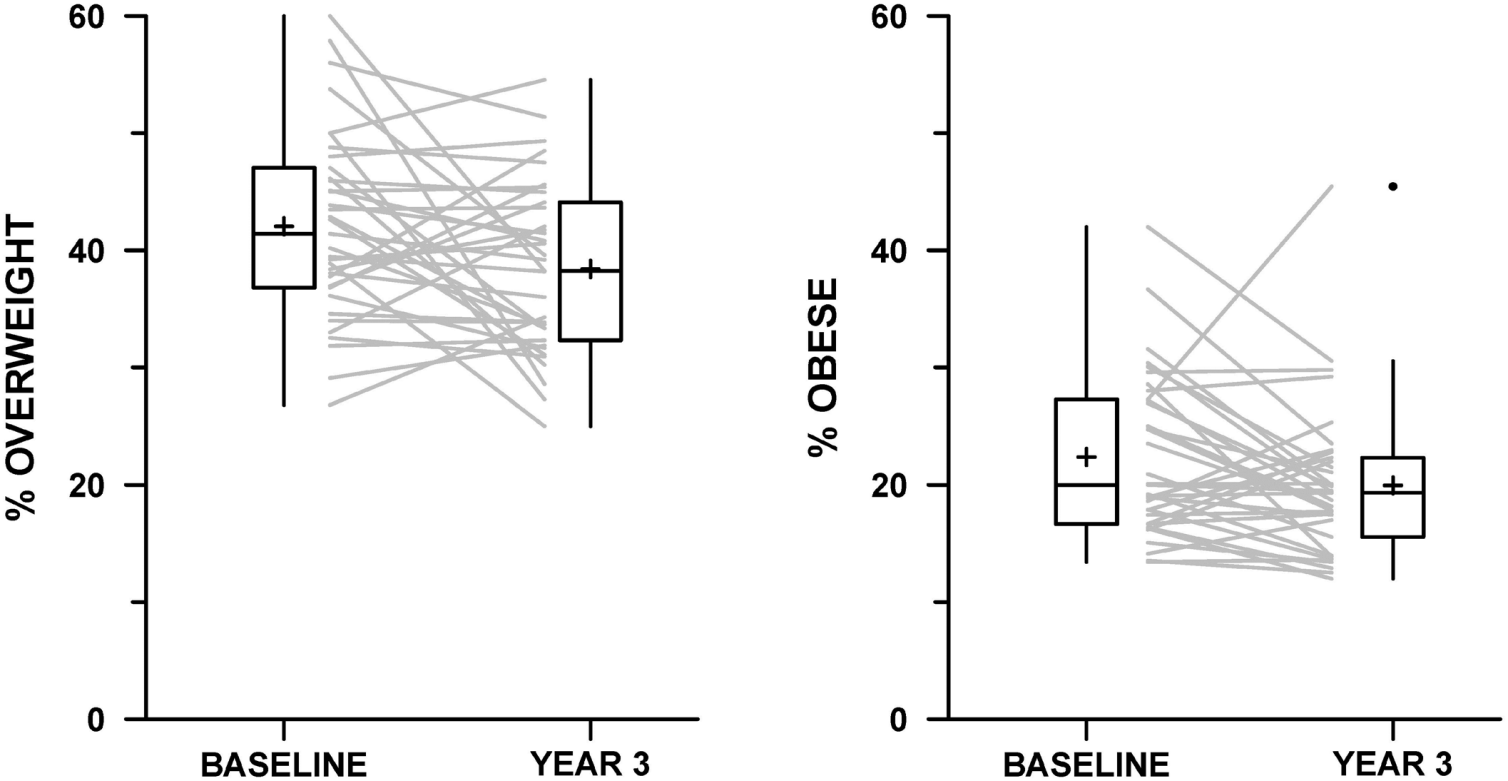
Change in school-level percent overweight and obese and percent obese from baseline to follow-up, Massachusetts Healthy Choices (n = 35).

Across schools, the percentage of students who reported awareness of the 5-2-1 message increased significantly from 14.5% to 41.5% (p < 0.001). The school-level means of 5-2-1 behaviors improved significantly over three years: FV consumption increased from 2.8 to 3.0 times daily (p = 0.0001), TV decreased from 2.2 to 2.1 hours daily (p = 0.008), and PA for ≥ 60 minutes increased from 3.7 to 3.8 days per week (p = 0.02). Total daily screen time did not change from baseline to follow-up (3.7 hours/day, SD 0.7). As shown in Table 2, the proportion across schools meeting goals for each of 5-2-1 behaviors targeted by the HC program increased significantly from baseline to follow-up. The mean percent of students achieving all three 5-2-1 behavioral goals increased from 5.6% to 7.0% (p = 0.0002). Among 35 schools with measured anthropometry on 7^th^ grade students, the mean percent overweight and obese decreased significantly from 42.1% to 38.4% (p = 0.016) and the percent obese fell from 22.4% to 20.0% (p = 0.045). Schools’ individual changes in prevalence of overweight and obesity are shown in Fig 1. Although one school shown on the spaghetti plot demonstrated a large increase in the percent obese from 6/22 (27%) to 10/22 (45%), this change was not statistically significant (Fisher’s exact test, p = 0.35) due to the small number of middle school students at this site.

**Table 2:** Percent across Schools Achieving Behavioral Goals^a^ and Percent Overweight and Obese at Baseline and 3-Year Follow-up^b^.

	*Baseline (Fall 2005)*			*Year 3 (Spring 2008)*			*Change*		
	Mean %	(SD)	[Range]	Mean	(SD)	[Range]	Adjusted Mean	(SE)	P value
**Behavioral Goals**									
**Fruits & vegetables** ^c^									
**% Achieving 5+ times/day**	16.4	(5.3)	[9.5–32.3]	19.4	(6.0)	[11.5–42.1]	2.96	(0.70)	0.0001
**Television viewing**									
**% Achieving** **≤** **2 TV hours/day**	53.4	(12.1)	[29.8–85.0]	58.2	(11.1)	[40.9–93.8]	4.75	(1.19)	0.0003
**Moderate-to-vigorous physical activity**									
**% Achieving 60+ min daily or on most days**	37.1	(6.5)	[21.7–53.0]	39.9	(9.0)	[21.2–62.2]	2.74	(1.17)	0.02
**% Achieving 5-2-1 goal (combined)**	5.6	(2.8)	[1.9–12.7]	7.0	(3.2)	[1.7–14.8]	1.42	(0.34)	0.0002
**Weight Status** ^d^									
**% Overweight and Obese**	42.1	(8.0)	[26.8–60.0]	38.4	(7.3)	[25.0–54.6]	-3.64	(1.43)	0.016
**% Obese**	22.4	(6.9)	[13.4–42.0]	20.0	(6.5)	[12.0–45.5]	-2.4	(1.16)	0.045

^a^ Percent of students in school achieving behavior, averaged across schools

^b^ Student’s paired t-test of significant difference from baseline to follow-up, adjusted for school % free and reduced price school lunch, % white, % female and mean age

^c^ Including 100% fruit juice; fruits and vegetables

^d^ n = 35 schools, 7th grade students only; overweight = BMI ≥ 85th percentile and < 95th percentile; obese = BMI ≥ 95th percentile of NCHS/CDC 2000 growth reference

The mean dose of HC intervention components met or exceeded minimum program requirements, but intensity ranged widely across schools, as shown in Table 3. On average, schools implemented 1.75 health promotions of the 5-2-1 message, 2.0 before- and after- school programs and 3.2 environmental and policy changes in Year 2. Sixty one percent of schools met the definition for the HC team. Mean expenditure of HC funds provided to schools was $6,145 (SD: $2,665). In a multivariable logistic regression model adjusting for baseline weight status and school characteristics, meeting the team definition was associated with a 0.83 lower odds (95% confidence interval (CI): 0.71–0.98) and the number of before- and after-school programs was associated with a 1.07 greater odds (95% CI: 1.01–1.12) of being overweight and obese.

**Table 3:** Distribution of Intervention Components implemented in Year 2 in Massachusetts Middle Schools Participating in the Healthy Choices Program (N = 35).

Intervention Components	Mean (SD)	Range
Number Planet Health Lessons taught	29.43	0–91
Number student lesson-exposures to Planet Health	960.93(1249)	0–6,059
Number Planet Health PE micro-units taught	5.98 (6.78)	0–22
Number teachers teaching Planet Health lessons	7.12 (5.95)	0–23
Number of partners outside school involved in HC activities	0.11 (0.45)	0–3
Number of school-wide promotions on 5, 2, 1	1.75(1.55)	0–5
Number of before- and after-school programs	2.02(1.68)	0–7
Number of environment and policy changes	3.21(1.81)	0–9

## Discussion

We evaluated a statewide, primary obesity prevention program comprising an interdisciplinary curriculum previously tested in an RCT [22, 23] and additional intervention components evaluated in non-experimental, participatory and qualitative research studies.[17, 24] We showed improvements over three years in the percent of students achieving 5-2-1 behavioral goals and reduced prevalence of overweight and obesity, adjusting for schools’ socio-demographic characteristics. To provide insights into how the program improved weight status, we modeled the contribution of HC components to variance in overweight and obesity. Overall, schools met or exceeded implementation guidelines, but only having a well-defined HC team was associated with decreased odds of overweight and obesity at follow-up.

School-level results of the HC evaluation were similar in magnitude to those found in the RCT of Planet Health curriculum [19].[22] We found a 3.6% decline in the prevalence of overweight and obesity over three school years. Among girls in Planet Health intervention schools participating in the RCT, prevalence of obesity defined as BMI and triceps skinfold over the 85^th^ percentile declined 3.3% conducted over two academic years and increased 2.2% among girls in control schools. We showed a mean, school-level increase in FV consumption of 0.2 times daily (p = 0.0001) over three years, smaller than an adjusted increase of 0.32 servings daily reported among girls in Planet Health schools. The decrease of -0.1 hours spent watching TV daily reported in HC was less than decreases found in the Planet Health RCT (girls: -0.6 hours; boys: -0.4 hours), but could be due to secular changes in TV viewing relative to computer use. The PA findings were difficult to compare because the RCT and HC evaluation had different units of measurement.

Prior research consistently has identified strong leadership as a key factor in institutionalization of school-based interventions.[33, 34] Results reported here and our baseline rapid assessment qualitative study also highlighted the importance of a team and providing opportunities for professional development and networking [17], as shown in other multi-component interventions targeting weight-related behaviors in schools.[35, 36] Chicago Public Schools fostered systems change in their Cool Foods initiative by using a task force to build networks to address resistance to change, different stakeholder priorities, lack of resources and institutional bureaucracy.[37] Regional HC coordinators also may have provided essential support for schools’ HC coordinators and teams and helped them bridge links among staff and external partners, consistent with pilot studies of the USDA’s Team Nutrition initiative [38] and qualitative studies of influences on adoption of changes in nutrition policies and environments in schools.[16, 39]

The HC before- and after-school program component was associated with a 7% increase in the odds of overweight and obesity. Our findings add to the still fairly limited information on before and after school programs. After-school programs have been more successful in increasing physical activity than in changing weight-related outcomes, with some exceptions.[40–43] HC schools designed their own before- and after-school programs that varied in the type of activities, intensity and reach, as shown in prior after-school obesity prevention efforts.[10] The cost of providing healthy snacks or other barriers also could have influenced adoption of before- and after-school activities addressing both diet and physical activity [43].[44]

We found no statistically significant associations with overweight and obesity for most HC program components, a finding that could be related to implementation dose, adequacy of resources and technical assistance or some other reason. HC schools were allowed flexibility to adopt different combinations of components as long as they adopted a minimum number within each component. The HC program guideline for minimum number of Planet Health lessons was half that tested in the Planet Health RCT, following schools’ preferences identified in a feasibility study of curriculum adoption.[24] Notably, HC schools on average taught 29 of 32 Planet Health lessons and 6 of 15 micro-units across 6^th^, 7^th^ and 8^th^ grades in Year 2, comparable with levels implemented annually across two grade levels in the RCT. The HC program captures several aspects of effective nutrition education and promotion outlined in USDA Team Nutrition initiative [45] including coherent, specific behavior-change messages across multiple components, ensuring intensity and duration of dose and providing professional networking for staff.

Findings of this study should be interpreted in light of several limitations. First, the evaluation relies on anonymous panel data, preventing examination of changes in individual students’ behaviors and weight status over the three-year intervention period. This study design is, however appropriate to Stage 5 dissemination research [20] and adapts methods for nutrition and obesity surveillance in schools that in combination with other evaluations [17, 22, 24, 26] can go beyond the RCT to satisfy needs for evidence to support for public health decision making.[19] Second, the absence of a control group makes it impossible to attribute the changes in 5-2-1 behaviors and weight status to the HC program. However, this systems-level intervention took place during a time when U.S. obesity rates were continuing to climb and just prior to national campaigns such as Let’s Move raised awareness of children’s weight-related behaviors.[46]. However, this program was implemented shortly after Congress passed the Child Nutrition and WIC Reauthorization Act of 2004 that mandated schools implement wellness policies beginning in 2006–2007. While many schools adopted Healthy Choices to meet this requirement, it is possible that some schools adopted other programs that could have contributed to our results. We were unable to evaluate cost-effectiveness of the HC program, important evidence for selecting which obesity prevention programs to implement more widely.[20, 21] An economic analysis based on the Planet Health RCT showed cost-effectiveness and cost-savings of providing schools with an annual stipend of $500 (1996 dollars) and reimbursing teachers for participating in training and wellness sessions.[26] On average, HC schools spent $6,145 of a $9,000 total stipend ($5,000 in Year 1; $3,000 in Year 2; and $1,000 in Year 3), but costs for technical assistance from HC regional coordinators and annual networking conferences were not estimated at the school level. Results of this study may only be generalizable to study populations coming from regions and schools with similar socio-demographic characteristics.

## Conclusions

We demonstrated improvements in nutrition and physical activity behaviors and weight status over three academic years in 45 middle schools implementing the HC program and found that a multidisciplinary HC team was central to schools’ efforts to reduce prevalence of overweight and obesity. Our findings are similar in magnitude to those reported in an earlier RCT of Planet Health, suggesting evidence-based interventions can be successfully adapted to real world settings when informed by a range of evaluation designs that ensure their relevance in different organizational contexts [16].[19]
